# False negative pericardial Focused Assessment with Sonography for Trauma examination following cardiac rupture from blunt thoracic trauma: a case report

**DOI:** 10.1186/s13256-015-0640-6

**Published:** 2015-07-15

**Authors:** Laura Baker, Ammar Almadani, Chad G. Ball

**Affiliations:** 1 McGill University, 845 Rue Sherbrooke Ouest, Montreal, Quebec H3A 0G4 Canada; 2 University of Calgary, Foothills Hospital, 1403 29 Street NW, Calgary, Alberta T2N 2T9 Canada; 3 University of Calgary, Foothills Medical Center, 1403 29 Street NW, Calgary, Alberta T2N 2T9 Canada

**Keywords:** Blunt trauma, Cardiac injury, Cardiac rupture, FAST exam, Pericardial laceration

## Abstract

**Introduction:**

The Focused Assessment with Sonography for Trauma examination is an invaluable tool in the initial assessment of any injured patient. Although highly sensitive and accurate for identifying hemoperitoneum, occasional false negative results do occur in select scenarios. We present a previously unreported case of survival following blunt cardiac rupture with associated negative pericardial window due to a concurrent pericardial wall laceration.

**Case presentation:**

A healthy 46-year-old white woman presented to our level 1 trauma center with hemodynamic instability following a motor vehicle collision. Although her abdominal Focused Assessment with Sonography for Trauma windows were positive for fluid, her pericardial window was negative. After immediate transfer to the operating room in the setting of persistent instability, a subsequent thoracotomy identified a blunt cardiac rupture that was draining into the ipsilateral pleural space via an adjacent tear in the pericardium. The cardiac injury was controlled with digital pressure, resuscitation completed, and then repaired using standard cardiorrhaphy techniques. Following repair of her injuries (left ventricle, left atrial appendage, and liver), her postoperative course was uneventful.

**Conclusions:**

Evaluation of the pericardial space using Focused Assessment with Sonography for Trauma is an important component in the initial assessment of the severely injured patient. Even in cases of blunt mechanisms however, clinicians must be wary of occasional false negative pericardial ultrasound evaluations secondary to a concomitant pericardial laceration and subsequent decompression of hemorrhage from the cardiac rupture into the ipsilateral pleural space.

## Introduction

Cardiac rupture following blunt thoracic trauma is associated with a high pre-hospital mortality and is therefore reported in less than 0.02% of patients admitted after blunt injury [1]. The dominant mechanics of most mediastinal injuries surround the sudden forceful deceleration of the heart in a space that is compressed between the sternum and vertebral column. Additional processes include increased intra-atrial pressure secondary to transmitted pressures from the abdominal or extremity veins; the evolution of myocardial contusion to necrosis followed by rupture; direct injury secondary to orthopedic injuries of the chest wall; and finally, injury resultant from lateralizing shear forces. The phase of the cardiac cycle at the moment of impact is also hypothesized to influence the severity of injury. Late diastole and early systole (that is, full chambers and closed valves) represent the phases with greatest potential for injury [1].

Cardiac tamponade is the most commonly observed manifestation of cardiac rupture. The diagnosis is often made in a persistently hypotensive patient with no identified source of hemorrhage [1]. Sonographic evaluation of pericardial fluid with right atrial or ventricular collapse is diagnostic [2]. Unfortunately, in occasional cases, an associated pericardial wall laceration prevents accumulation of intra-pericardial fluid. Consequently, these patients generate a hemothorax in the ipsilateral pleural space [1].

The Focused Assessment with Sonography for Trauma (FAST) was first introduced in 1996 as a diagnostic modality to detect free fluid within the peritoneal and pericardial spaces [3]. Its rapid incorporation into the secondary survey modified the standard of care and was dominantly based upon an impressive reported performance (sensitivity = 100%; specificity = 97%; accuracy = 97%; positive predictive value = 81%; negative predictive value = 100%) for detecting cardiac rupture in typical trauma scenarios [4]. Unfortunately, our group also highlighted a series of cardiac ruptures following penetrating trauma that were occult to repeatedly normal pericardial windows on FAST, as well as to formal echocardiography [5]. Intraoperative findings in all patients revealed cardiac injuries with concurrent lacerations of the pericardial sac, allowing for decompression of the hemopericardium into the ipsilateral thoracic cavity [5]. Another evaluation of 58,304 trauma admissions over a 5-year period in a tertiary care center reported two cases of blunt cardiac rupture with associated pericardial lacerations. Cardiac injury was diagnosed by FAST in one of the two cases. The other patient underwent an exploratory thoracotomy, revealing a left ventricular perforation with associated pericardial wall laceration. This patient died intraoperatively [1].

We report a case that documents the only reported survivor following blunt cardiac rupture with an associated pericardial laceration in the context of a false negative pericardial window on FAST examination.

## Case presentation

A fit, active, and otherwise healthy 46-year-old white woman presented to our level 1 trauma center with hemodynamic instability (systolic blood pressure = 82mmHg; heart rate = 144) and hypoxia following a motor vehicle collision at highway speeds. Her injuries initially included multiple bilateral rib fractures and a massive left hemothorax. The FAST examination was positive in the intra-abdominal windows, but negative within the cardiac window. At our center, all FAST examinations are performed by experienced trauma surgeons who have long-term experience in both the performance and advancement of ultrasonography in trauma. Bilateral chest tubes were inserted, followed by the evacuation of 1.1 liters of blood from her left hemithorax. She remained persistently hypotensive and tachycardic despite resuscitation with the massive transfusion protocol (total: 8 units of red blood cells; 7 units of fresh frozen plasma). After persistent instability despite 2 units of red blood cells, the critically ill patient was transferred to the operating suite.

Exploration of her peritoneal cavity revealed a moderate liver laceration which was packed, and eventually underwent sutured hepatorrhaphy. Immediately after assessing that the majority of blood loss was clearly not within the peritoneal cavity, she underwent a left lateral thoracotomy. Time from the abdominal incision to completion of the thoracotomy was 4 minutes. This exploration revealed a 3cm laceration in the left pericardium with an underlying 2cm rupture of the left ventricle. A 1cm laceration of the left atrial appendage was also identified. Digital occlusion to arrest all ongoing hemorrhage was utilized until resuscitation was nearly complete. The atrial appendage injury was stapled with a TX-30 stapler, while the left ventricular injury was repaired with two 4–0 prolene sutures on SH needles. Intraoperative as well as postoperative echocardiography confirmed the absence of synchronous intracardiac valvular or other injuries.

## Discussion

The critical ‘take-home’ message of this report remains highlighting the atypical, but real limitation of cardiac views within the FAST examination. Although the majority of the published literature discussing the rare occurrence of false negative cardiac windows highlights penetrating mechanisms with right-sided, low pressure cardiac injuries concurrent to an ipsilateral pericardial laceration, this report discusses a blunt etiology. It stresses the importance of considering an underlying cardiac injury in the event of a negative pericardial window even in the presence of a blunt mechanism. It also re-emphasizes the reality that the single limitation (beyond inadequate views secondary to subcutaneous emphysema) of the pericardial FAST examination occurs in cases of small cardiac lacerations and concurrent pericardial defects. As discussed, these patients also typically possess a synchronous hemothorax that remains partially undrained despite tube thoracostomy. As a result, any patient who sustains penetrating (and in rare cases blunt) thoracic injury in the context of a persistently undrained hemothorax must undergo urgent evaluation to further delineate the possibility of a missed cardiac injury (that is, false negative pericardial window).

It should also be noted that although FAST examinations pose significant benefit over cross-sectional imaging with computed tomography (CT) due to their utility in patients with hemodynamic instability, it is possible to diagnose cardiac rupture via CT using intravenous contrast medium [6, 7]. Unfortunately, this modality was not appropriate in our case given her persistent instability and critical illness.

## Conclusions

Despite the extreme rarity of surviving to present at a trauma center, the occasional arrival of a patient with blunt thoracic trauma and a concurrent cardiac rupture, mandates a prepared clinician who has the ability to rapidly diagnose and treat this life-threatening scenario. The FAST pericardial window represents the workhorse of achieving this diagnosis and remains sensitive and accurate in experienced hands. Exceptions, as indicated by this report, include arriving at a false negative diagnosis in the context of a concurrent pericardial laceration and decompression into the ipsilateral pleural space causing a hemothorax. The diagnosis of a true cardiac laceration/rupture must be contemplated when tube thoracostomy insertion does not clear all hemorrhage from the chest, and/or when drainage from the tube persists.

In summary, even a test with a reported high sensitivity and accuracy such as the FAST examination must not limit clinical acumen in less common scenarios. Although the pericardial FAST window continues to perform extremely well in the context of most trauma patients, the observed failures among patients who lack a closed pericardial sack (that is, the ability to generate a collection of detectable fluid) must remain at the forefront of the ultrasonographer’s mind when assessing critically injured patients.

## Consent

Written informed consent was obtained from the patient for publication of this case report. A copy of the written consent is available for review by the Editor-in-Chief of this journal.
